# Genome-wide SSR marker development and application in molecular breeding of *Hemerocallis*

**DOI:** 10.3389/fpls.2026.1740316

**Published:** 2026-03-25

**Authors:** Feifan Hou, Xinjuan Guo, Xinyan Jia, Mingyuan Hu, Jie Su, Xufei Liang, Yuqian Qin, Yang Gao, Guoming Xing, Sen Li

**Affiliations:** 1 Shanxi Key Laboratory of Germplasm Resources Innovation and Utilization of Vegetable and Flower, College of Horticulture, Shanxi Agricultural University, Taigu, China; 2 Datong Daylily Industrial Development Research Institute, Datong, China

**Keywords:** genetic diversity analysis, genetic map, genome-wide, *hemerocallis*, molecular breeding, SSR markers

## Abstract

The *Hemerocallis* genus belongs to the *Asphodelaceae* family and is a perennial herbaceous plant. To facilitate molecular breeding and industrial development in *Hemerocallis*, this study developed genome-wide simple sequence repeat (SSR) markers based on the genome of *Hemerocallis* ‘Meng Zi Hua’. Two independent marker sets were applied for germplasm genetic diversity analysis and genetic linkage map construction. A total of 913,004 SSR loci were identified, with dinucleotide and mononucleotide repeats being the dominant types and AT-rich motifs being the main repeat units. From the first set of 48 randomly selected SSRs, 32 demonstrated polymorphism when validated across six germplasm accessions. These 32 polymorphic markers were subsequently employed to analyze a panel of 287 *Hemerocallis* germplasms. The analysis revealed high polymorphism with an average polymorphic information content (PIC) value of 0.7371. The germplasms were classified into six groups via unweighted pair group method with arithmetic mean (UPGMA) clustering and inferred to comprise two distinct gene pools based on population structure analysis. The second set comprised 156 SSRs, of which 153 produced clear amplification products. Among these, 90 SSRs showed parent-progeny polymorphism and were used to construct a genetic linkage map. This genetic map contained 90 markers distributed across 11 linkage groups with a total map length of 3051.49 cM. Additionally, five quantitative trait loci (QTLs) associated with petal color traits were detected and explained 8.41% to 38.82% of phenotypic variation. These findings enrich molecular marker resources available, and establish a technical foundation for future marker-assisted breeding and genetic improvement for *Hemerocallis*.

## 1 Introduction

The *Hemerocallis* genus belongs to the *Asphodelaceae* family and is a herbaceous perennial of global importance. They are widely cultivated for their horticultural value and ecological adaptability, holding significant economic potential. The genus exhibits substantial phenotypic diversity in traits such as flower color, shape, and blooming period. This rich phenotypic diversity provides a foundation for genetic improvement and cultivar development (Gao Y, et al., 2025; Sun et al., 2025). However, the effective utilization of these germplasm resources has been hampered by a scarcity of efficient molecular markers necessary for accurate genetic characterization, diversity assessment, and the construction of core germplasm collections.

Recent advances in high-throughput sequencing and the declining cost of genome-wide sequencing have facilitated the large-scale development of SSR markers in plants. SSRs are valued for their high polymorphism, specificity, and reproducibility, and have been widely used to identify germplasm resources across numerous species, including *Primula denticulata* (Yadav et al., 2024), *Argania* sp*inosa* (Rabeh et al., 2024), *Curcuma alismatifolia* (Ye et al., 2024), *Solanum lycopersicum* (Castellana et al., 2020), *Trifolium repens* (Ma S, et al., 2020), *Populus* (Sun et al., 2020), and *Camellia sinensis* (Liu et al., 2018). Specific applications demonstrate their utility. For genetic diversity assessment, polymorphic SSR markers have effectively differentiated accessions of grain amaranth (Vats et al., 2023), pigeonpea (Kimaro et al., 2020), and chrysanthemum (Luo et al., 2019), the latter also revealing associations with petal morphology. In marker development, genome-wide scans in sugarcane (Xu et al., 2025) and melon (Zhu et al., 2016) have identified abundant SSR loci, with melon studies further applying these markers for comparative mapping with related species. These examples underscore the role of SSR markers in facilitating germplasm analysis and genomic research.

In germplasm resource identification, a fundamental challenge is the accurate differentiation of crop varieties. The development of PCR-based molecular markers has greatly advanced DNA fingerprinting techniques. Among these, SSR markers are now a well-established tool for germplasm resources identification and have been widely applied across diverse plant species (Tzuri et al., 1991; Santosh et al., 2022; Pei et al., 2010; Liang et al., 2020). For instance, only five SSR markers were needed to differentiate 33 grapevine cultivars (Thomas et al., 1994). Similarly, a combination of four genomic SSR (gSSR) and three expressed sequence tag SSR (EST-SSR) markers. generated fingerprints for 203 sweet potato varieties (Meng et al., 2018), and just three SSR markers enabled complete discrimination among 10 white clover varieties (Ma S, et al., 2020). By detecting polymorphism at the DNA level, the fingerprinting technique based on molecular markers offers high accuracy, reliability, operational simplicity, and reproducibility. These advantages make DNA fingerprinting an effective tool for the precise identification of crop germplasm resources.

A genetic linkage map illustrates the relative positions and distances between genes or markers on a linkage group. The construction of genetic linkage map is one of the core components of genetic research. A high quality genetic map with a large number of polymorphic markers evenly distributed across the linkage groups provide an essential framework for QTL mapping, marker-assisted selection (MAS), gene cloning, and comparative genomics (Jiang et al., 2024). Due to their high polymorphism and reproducibility, SSR markers remain a preferred choice for constructing genetic maps in many crop species (Liu et al., 2024). In horticultural research, genetic maps have been widely employed by many researchers to locate various plant traits. For instance, in *Setaria japonica*, a genetic map comprising 167 SSR markers across 27 linkage groups (total length of 2069 cM) was constructed (Wang et al., 2023). Similarly, an intraspecific genetic map for *Hemerocallis citrina* was constructed using 124 EST-SSR markers, spanning 1535.07 cM across 11 linkage groups (Hou et al., 2025). In *Setaria italica*, a genetic map with 215 markers (213 SSR markers and 2 insertion-deletion markers) facilitated the identification of 46 QTLs for twelve agronomic traits, which included 13 major effect QTLs (Gao L, et al., 2025). Likewise, a major QTL of anthracnose resistance was detected and validated on the genetic linkage map over two years in tea plant (Zhang et al., 2025).

Despite the widespread use of SSR markers in other plant species and the early-stage genetic mapping efforts in *Hemerocallis*, genome-wide SSR marker development and their systematic application in germplasm diversity analysis and trait-related QTL mapping remain limited. This gap hinders the in-depth exploration and utilization of genetic resources in this genus. This study focuses on the genome-wide SSR markers development, genetic diversity assessment of germplasm resources, genetic linkage map construction of hybridization population and QTL mapping analysis in the *Hemerocallis* genus. The research aims to enhance the utilization of germplasm resources, facilitate MAS breeding, and provide a robust technical foundation for further research genetic breeding studies in *Hemerocallis*.

## 2 Materials and methods

### 2.1 Plant materials

A total of 287 *Hemerocallis* germplasm accessions were randomly selected from the germplasm repository at Shanxi Agricultural University for initial evaluation of the developed SSR markers (**Supplementary Table 1**). The germplasm repository is located in the Taigu District, Jinzhong City, Shanxi Province (37°25′N, 112°35′E), where all plants were cultivated under standard management and maintained in healthy condition. In 2018, an F_1_ hybridization population of 390 progenies was generated by *Hemerocallis* ‘Liu Yue Hua’ (female parent, monochromatic flower) and *Hemerocallis* ‘Frans Hals #1’ (male parent, bicolor flower) as cross parents. This population exhibited extensive variation in petal color. From this F_1_ population, a subset of 116 progenies was randomly selected to construct the genetic linkage map. The genome-wide sequence of *Hemerocallis* ‘Meng Zi Hua’ was obtained from GenBank (GCA_017893485.1, ASM1789348v1; https://www.ncbi.nlm.nih.gov/assembly/GCA_017893485.1). The resequencing data for *Hemerocallis* ‘Liu Yue Hua’ and *Hemerocallis* ‘Frans Hals #1’ were published on the Genome Sequence Archive (GSA: CRA037226; https://ngdc.cncb.ac.cn/gsa/browse/CRA037226).

### 2.2 DNA extraction and detection

Young leaf tissue (approximately 0.2 g) was collected from each of the 287 germplasm accessions and 116 F_1_ hybrid progenies into pre-labeled 2 mL centrifuge tubes. The tubes were immediately placed on ice. Two steel beads were added to each tube, which was then flash-frozen in liquid nitrogen for precooling. The frozen tissue was homogenized using a tissue grinder for 2 min. Genomic DNA was extracted using a modified CTAB method (Paterson et al., 1993). The extracted DNA was dissolved in 100 μL of double-distilled water (ddH_2_O). DNA quantity and quality were measured using a NanoDrop ND-2000C Spectrophotometer (Thermo Fisher Scientific Inc., Waltham, MA, USA). Samples with acceptable quality were diluted with ddH_2_O to a working concentration of 50 ng/μL and stored at -40 °C for downstream applications. The results of agarose gel electrophoresis for the DNA samples are shown in **Supplementary Figure 1**.

### 2.3 Identification and development of genome-wide SSRs of *Hemerocallis* ‘Meng Zi Hua’

The SSR loci were identified from the *Hemerocallis* ‘Meng Zi Hua’ genome using the MISA software. The parameters in MISA were set as follows: a minimum of 10 repeats for mononucleotide SSRs, 6 repeats for dinucleotide SSRs, and 5 repeats for tri-, tetra-, penta-, and hexanucleotide SSRs. The preliminary results from MISA were further processed with Bedtools. Finally, the distribution and frequency of the identified SSRs were statistically analyzed, including the total number and proportions of different repeat motifs. SSR primers were designed using Primer3.0 software and named according to the ‘sau + number’ format. For genetic diversity assessment, six *Hemerocallis* accessions were used as PCR templates to identify the polymorphism markers from 48 SSRs. Ultimately, 32 polymorphic SSR markers were randomly selected and synthesized (see **Supplementary Table 2** for details). For genetic linkage map construction, electronic PCR was conducted between the two hybrid parents (‘Liu Yue Hua’ and ‘Frans Hals #1’) to screen polymorphic SSRs, and then 156 polymorphic SSRs were randomly selected for synthesis. These SSRs were then validated by performing PCR amplification using genomic DNA from the two parents and six F_1_ progenies exhibiting distinct petal colors, confirming the polymorphism of SSRs in both parents and progenies. Finally, the validated polymorphic SSRs were used to genotype 116 F_1_ progenies from the hybridization population of ‘Liu Yue Hua’ and ‘Frans Hals #1’, and the electrophoretic band patterns were recorded in detail.

### 2.4 SSR-PCR amplification reaction and detection of polyacrylamide gel electrophoresis

PCR was performed in a 10 µL reaction mixture containing 3 µL of 2×Taq PCR Master Mix (5 U/μL, Real-Times Biotechnology Co., Ltd., Beijing, China), 2 µL of template DNA, 0.5 µL each of forward and reverse primers (0.2 µM final concentration each), and 4 µL of ddH_2_O. The amplification protocol consisted of an initial denaturation at 94 °C for 3 min; followed by 24 cycles of denaturation at 94 °C for 45 s, annealing at 58 °C for 1 min, and extension at 72 °C for 1 min; with a final extension at 72 °C for 5 min. PCR products were measured on a 30% nondenaturing polyacrylamide gel and visualized by silver staining. Banding patterns were scored according to the method described by Hou et al. (2025).

### 2.5 Group structure analysis

Genetic diversity among the 287 *Hemerocallis* accessions was assessed using SSR markers. The amplified fragments were scored as a binary matrix (1 for band presence, 0 for band absence, with invalid results coded as 9). This matrix was used for subsequent analyses. Basic genetic parameters of 287 *Hemerocallis* accessions, including the observed number of alleles (Na), effective number of alleles (Ne), expected heterozygosity (H), Shannon’s information index (I), and the polymorphic band ratio, were calculated using Popgene 32 software. The PIC for each SSR marker was determined using Powermarker v3.25. Based on the binary matrix, genetic distance between 287 accessions were calculated with NTSYS software. A dendrogram was constructed via the UPGMA using the Tree Plot program and visualized in MEGA11 software. Population genetic structure was analyzed using STRUCTURE version 2.3.4, with the putative number of populations (K) set from 1 to 10 (five iterations per K). The optimal K value was determined based on the ΔK statistic, and corresponding genetic structure visualizations were generated using TBtools software. To achieve unambiguous discrimination of all 287 *Hemerocallis* accessions, a core set of markers was selected by sequentially adding markers in descending order of PIC values until complete differentiation was attained. The corresponding binary data for the selected core markers were then compiled using Microsoft Excel for identification purposes.

### 2.6 Genetic linkage map construction

A genetic linkage map was constructed using GACD software (Version 1.2). For the grouping stage, the logarithm of the odds ratio (LOD) threshold method (initial LOD = 3) was adopted, and markers were clustered into 11 linkage groups based on genetic distance. During the ordering process, the k-Optimality algorithm (with recombination frequency as the reference index) was employed, combined with the 2-OptMAP ordering strategy and Shortest NN neighbor search (threshold = 10) to finalize marker ordering. The marker positions were subsequently optimized using a Ripple operation with a window size of 5. The Primer information was shown in **Supplementary Table 5.**

### 2.7 Determination of petal color traits in hybridization population

During the full flowering period from June to August 2022, fresh flowers were collected daily within two-time windows: 6:00-8:00 and 21:00-24:00. Upon collection, the colors of the inner and outer petals from parental plants and F_1_ population were measured immediately using a CM-700d spectrophotometer (KONICA MINOLTA Investment Ltd, Shanghai, China). Measurements were conducted under a D65 light source to obtain the lightness (L*), redness (a*), and yellowness (b*) values. For each F_1_ progeny, three fully open flowers were sampled. Given that each flower possesses three inner and three outer petals, the average values for each petal type from per progeny were calculated and used to represent the inner and outer petal colors, respectively. The complete phenotypic dataset for petal color in the hybrid population has been published previously (Qin et al., 2023).

### 2.8 QTL mapping of petal color

The QTL analysis for petal color was conducted on the F_1_ population using interval mapping method in GACD software. A permutation test with 1,000 replicates was applied to determine the genome-wide LOD significance threshold, and the genome was scanned at 1 cM intervals. The *qOPL1.1* denotes the first QTL detected on linkage group 1 associated with the L* value of the outer petal.

## 3 Results

### 3.1 Development of genome-wide SSR markers based on *Hemerocallis* ‘Meng Zi Hua’

The genomic sequences of *Hemerocallis* ‘Meng Zi Hua’ were analyzed using MISA software. A total of 734 sequences, comprising 3,775,579 kb, were detected, from which 913,004 SSR loci were identified, with an average density of one SSR locus per 4.14 kb. All six repeat motifs (mononucleotide to hexanucleotide) were detected in the *Hemerocallis* genome (**Table 1**). Mononucleotide and dinucleotide repeats were the most abundant, accounting for 30.39% and 42.12% of the total SSRs, respectively. Trinucleotide repeats accounted for 203,827 loci (22.32%), whereas tetra-, penta-, and hexanucleotide repeats were rare, together representing only 5.14% of the total. Thus, the frequency of SSR types decreased with increasing repeat motif length. Dinucleotide SSRs showed the smallest average distribution distance (9.82 kb), while hexanucleotide SSRs had the largest (765.37 kb), a value 77.94 times that of dinucleotide SSRs. Overall, with the exception of the dinucleotide motif, the average distance between SSR loci increases as the number of nucleotides per repeat motif increases.

**Table 1:** Distribution of SSR repeat motifs in the genome of *Hemerocallis* ‘Meng Zi Hua’.

Repeat type	Count	Proportion in all SSRs(%)	Average distance/kb
Mononucleotide	277,448	30.39	13.61
Dinucleotide	384,559	42.12	9.82
Trinucleotide	203,827	22.32	18.52
Tetranucleotide	33,973	3.72	111.13
Pentanucleotide	82,64	0.91	456.87
Hexanucleotide	49,33	0.54	765.37
Total	913,004	100	4.14

**Figure 1:**
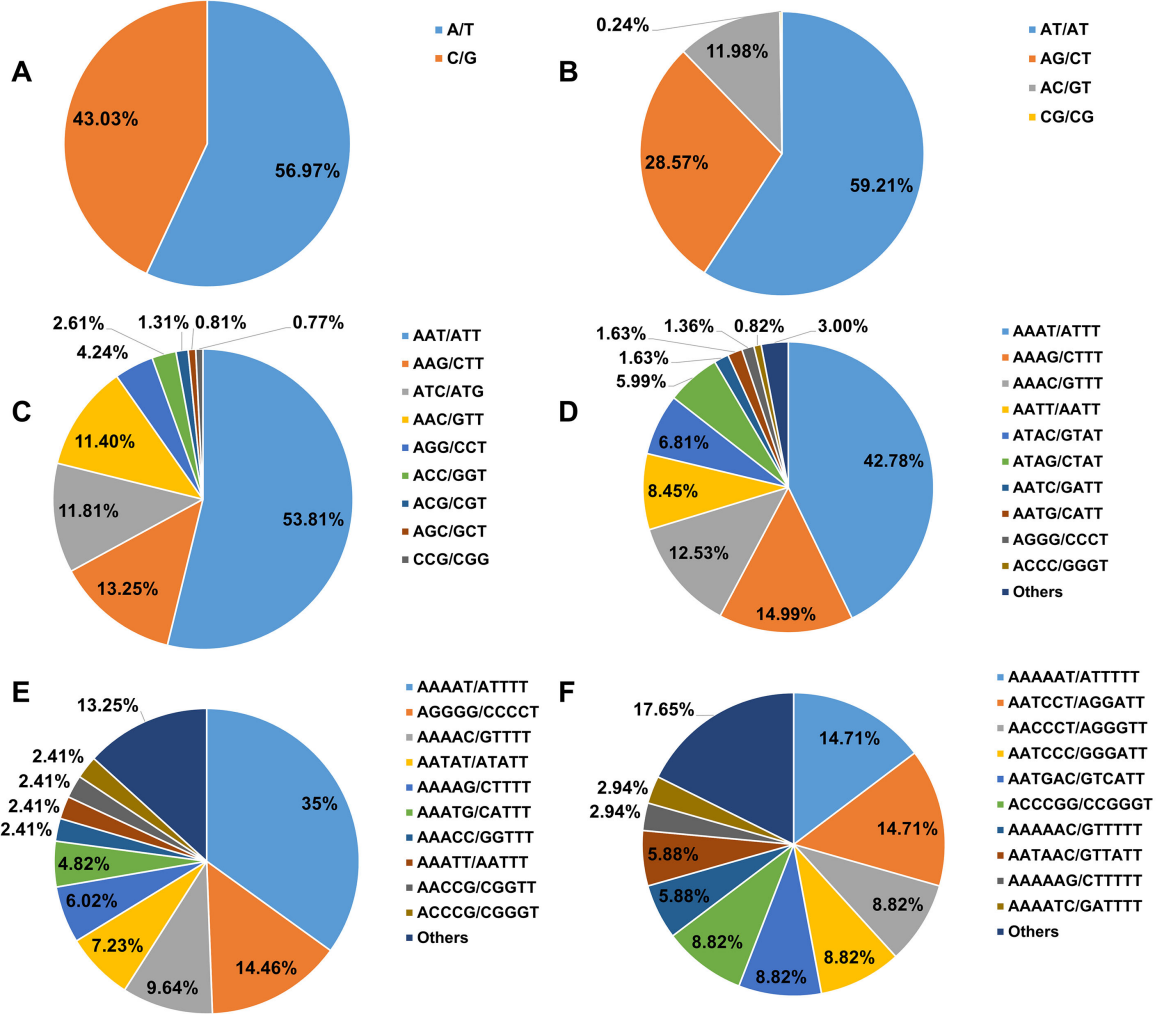
Distribution characteristics of SSR repeat motifs in genome *Hemerocallis* ‘Meng Zi Hua’. **(A)** Mononucleotide repeats; **(B)** Dinucleotide repeats; **(C)** Trinucleotide repeats; **(D)** Tetranucleotide repeats; **(E)** Pentanucleotide repeats; **(F)** Hexanucleotide repeats.

**Figure 2:**
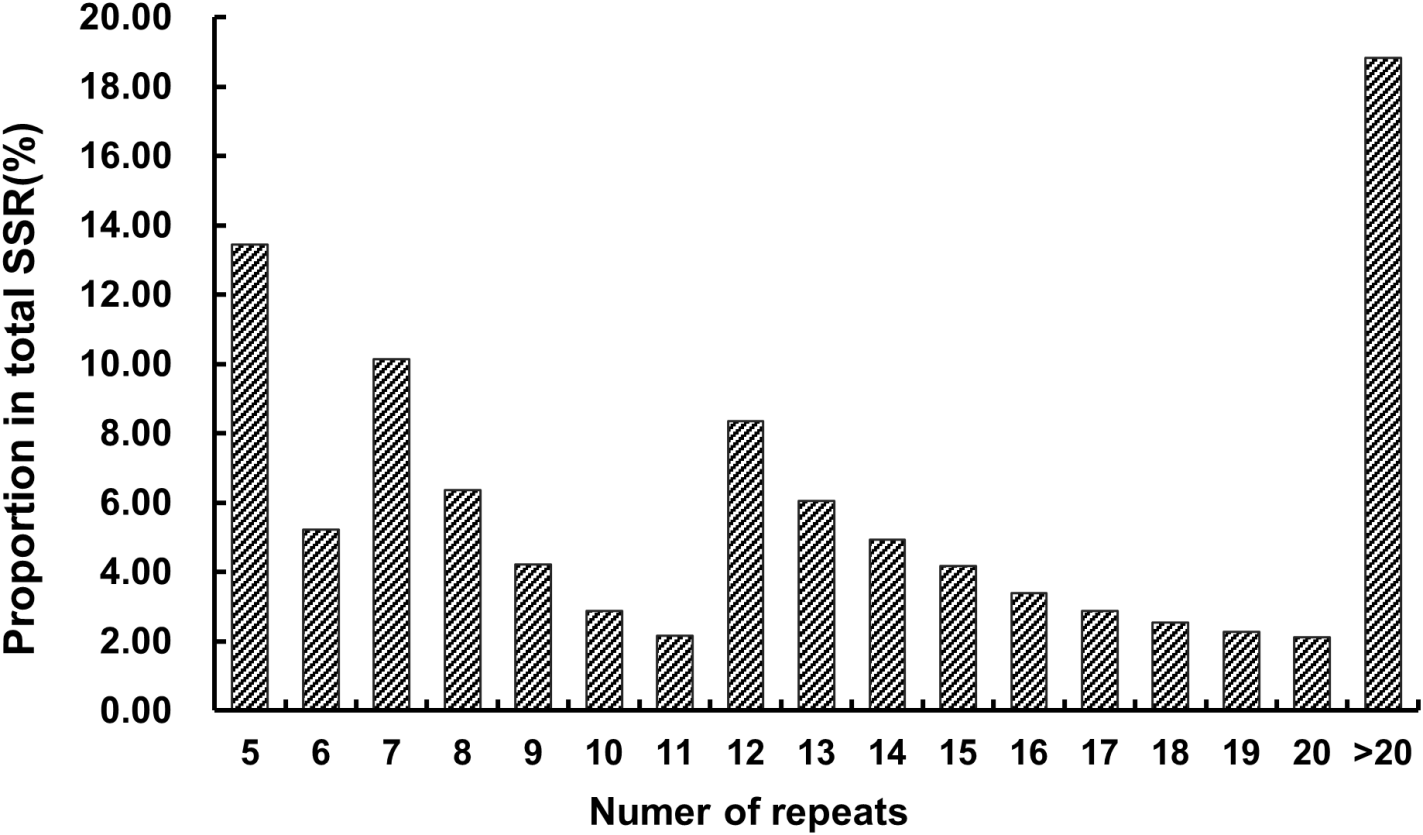
Frequency distribution of repeat number of SSRs in *Hemerocallis* genome.

The genome of *Hemerocallis* ‘Meng Zi Hua’ exhibited considerable diversity in SSR motif types, with 328 distinct repeat units identified (**Figure 1**). Hexanucleotide repeats showed the highest diversity (209 motif types), whereas mononucleotide repeats showed the lowest (only 2 motif types). Dinucleotide, trinucleotide, tetranucleotide, and pentanucleotide repeats contained 4, 9, 28, and 76 unique motif types, respectively. The A/T motif was predominant, accounting for 56.97% of mononucleotide repeat SSRs and 17.33% of total SSRs. Among dinucleotide repeats, the AT/AT motif was predominant (59.21% of dinucleotide repeat SSRs, 25.01% of total SSRs). For tri-, tetra−, penta−, and hexanucleotide repeats, the AAT/ATT (53.81% of trinucleotide repeat SSRs), AAAT/ATTT (42.78% of tetranucleotide repeat SSRs), AAAAT/ATTTT (34.94% of pentanucleotide repeat SSRs), and AAAAAT/ATTTTT (14.71% of hexanucleotide repeat SSRs) motif were the most frequent, respectively. Notably, all these dominant motifs types were highly enriched in adenine (A) and thymine (T) bases. The repeat numbers of different SSR motifs were summarized in **Table 2** and **Figure 2**. Among SSRs with fewer than 20 repeats, those containing 5, 7, and 12 repeats exhibited the highest proportions, accounting for 13.45%, 10.14%, and 8.35% of all SSRs, respectively.

**Table 2:** Statistics of repetition numbers of different SSR repeat motifs in the genome of *Hemerocallis* ‘Meng Zi Hua’.

SSR repeat types	Number of repeats																	Total number	Proportion (%)
	5	6	7	8	9	10	11	12	13	14	15	16	17	18	19	20	>20 repeats		
Mononucleotide	ND	ND	ND	ND	ND	ND	ND	60149	40889	32081	25811	19171	14784	11859	9599	8535	54570	277448	30.39
Dinucleotide	ND	ND	70148	46204	30461	20430	15053	12334	11010	10279	9977	9697	9652	9660	9761	9680	110213	384559	42.12
Trinucleotide	97405	36937	17141	9586	6713	5115	4290	3550	3134	2638	2312	1999	1825	1596	1345	1182	7059	203827	22.32
Tetranucleotide	17135	7948	4273	1927	1023	566	356	204	143	91	67	42	36	28	28	15	91	33973	3.72
Pentanucleotide	5146	1801	621	303	171	77	50	29	16	11	8	3	6	4	2	1	15	8264	0.91
Hexanucleotide	3105	1081	414	154	86	32	17	10	8	4	5	2	2	1	1	0	11	4933	0.54
Total	122791	47767	92597	58174	38454	26220	19766	76276	55200	45104	38180	30914	26305	23148	20736	19413	171959	913004	100.00
Proportion of each repeat number (%)	13.45	5.23	10.14	6.37	4.21	2.87	2.16	8.35	6.05	4.94	4.18	3.39	2.88	2.54	2.27	2.13	18.83	100	

ND indicates that this type of repetition was not detected in the genome.

### 3.2 SSR primer design and polymorphism detection

Primers were designed using Primer3 software based on the flanking sequences of SSR loci identified by MISA, yielded a total of 634,093 primer pairs of SSRs. Statistical analysis of SSR primers derived from the *Hemerocallis* ‘Meng Zi Hua’ genome showed that primers from dinucleotide repeat SSRs were the most abundant (43.15% of all developed SSR primers), followed by primers from mononucleotide repeat SSRs (35.54% of all developed SSR primers). On average, the SSR primers had a density of one primer per 16.75 kb across the genome (**Table 3**). Simulated PCR amplification using the re-sequencing data of *Hemerocallis* ‘Liu Yue Hua’ and *Hemerocallis* ‘Frans Hals #1’ revealed that 20,285 primers produced a single amplicon in the *Hemerocallis* ‘Liu Yue Hua’ genome, while 6,820 primers did so in the *Hemerocallis* ‘Frans Hals #1’genome. Among these, 1,755 primers successfully amplified a single product in both genomes, and 1,117 primers exhibited polymorphisms between the two germplasm accessions, with amplicon size differences ranging from 1 to 195 bp.

**Table 3:** Statistics and distribution of repeat motifs of developed SSR primers in the genome of *Hemerocallis* ‘Meng Zi Hua’.

Repeat type	Count	Proportion in all developed primers of SSRs (%)	Average distance/kb
Mononucleotide	225,364	35.54	16.75
Dinucleotide	273,594	43.15	13.79
Trinucleotide	102,666	16.19	36.76
Tetranucleotide	22,323	3.52	169.07
Pentanucleotide	6,553	1.03	575.94
Hexanucleotide	3,592	0.57	1,050.70
Total	634,093	100.00	16.75

**Figure 3:**
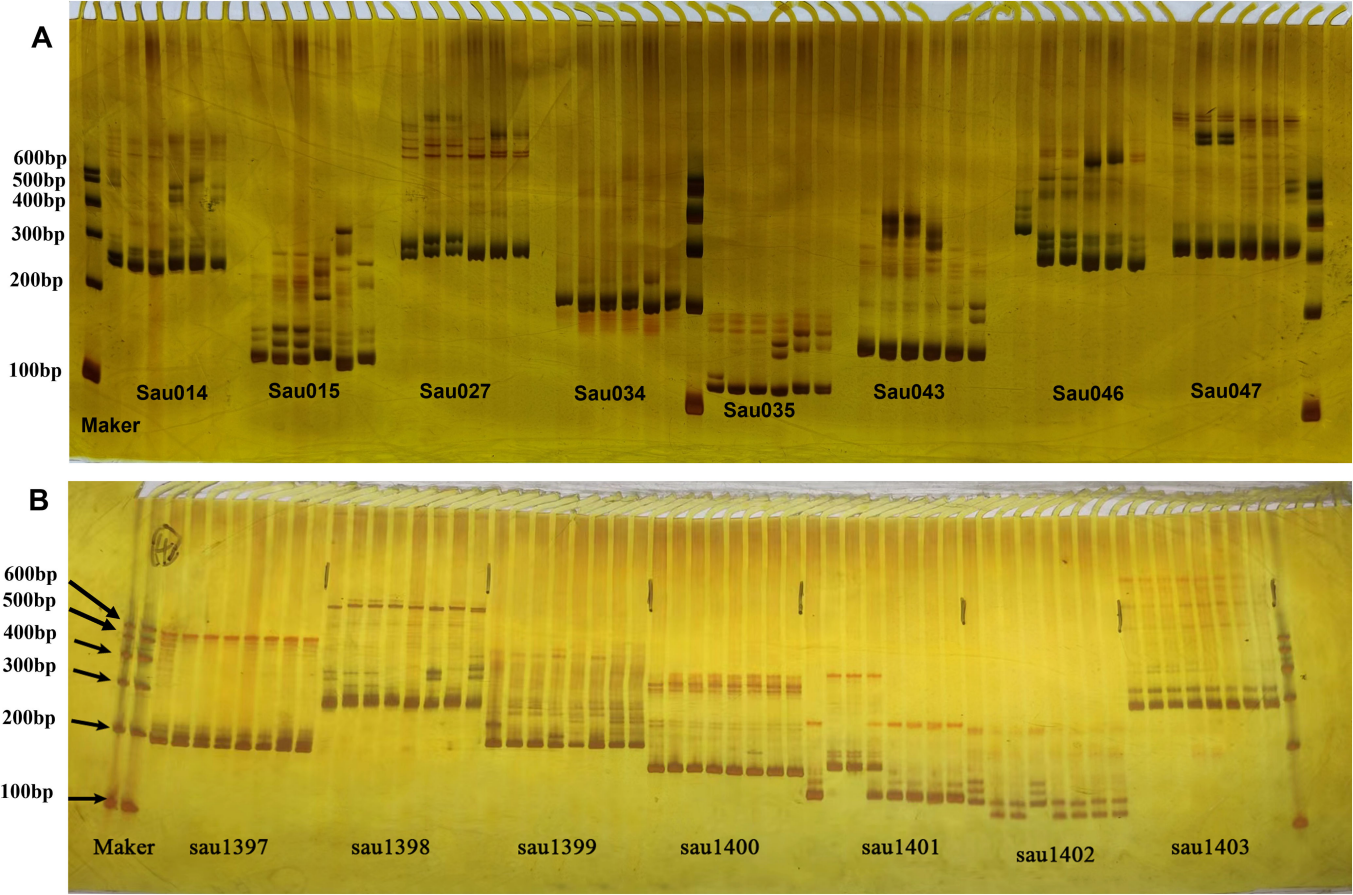
Polyacrylamide gel electrophoresis of polymorphic SSR loci. **(A)** Polymorphism of 8 SSR markers in 6 *Hemerocallis* germplasms for genetic diversity analysis; **(B)** Polymorphism of 7 SSR markers in parents and their F_1_ progenies for genetic linkage map construction.

Electronic PCR identified 1,117 polymorphic SSR primers between *Hemerocallis* ‘Liu Yue Hua’ and *Hemerocallis* ‘Frans Hals #1’. In these markers, 48 were randomly selected for polymorphism validation across six *Hemerocallis* germplasms accessions, including *Hemerocallis* ‘Liu Yue Hua’, *Hemerocallis* ‘Frans Hals #1’, *Hemerocallis* ‘Lullaby Baby #1’, *Hemerocallis* ‘Golden Doll #1’, *Hemerocallis* ‘Datong Huang Hua #1’ and *Hemerocallis* ‘Chong Li Hua #1’ (**Figure 3**). Polyacrylamide gel electrophoresis analysis revealed that 32 of the 48 tested SSRs produced clear bands. In parallel, 156 SSR primers were randomly synthesized and amplified using DNA from parents of *Hemerocallis* ‘Liu Yue Hua’ and *Hemerocallis* ‘Frans Hals #1’, and 116 F_1_ progenies. In these primers, 153 generated clear amplification products, and 90 exhibiting parent-progeny polymorphism (**Figure 3**).

### 3.3 Analysis of genetic diversity of *Hemerocallis* germplasm accessions based on SSR markers

Genetic diversity analysis was conducted on 287 *Hemerocallis* germplasm accessions using 32 SSR markers (**Supplementary Table 3**). The polyacrylamide gel electrophoresis revealed that these markers amplified a total of 247 bands, with an average of 7.72 bands per marker. Analysis with Popgene software revealed 62.76 alleles and 51.52 effective alleles in total, corresponding to an average of 1.96 alleles and 1.61 effective alleles per marker. The expected heterozygosity (H) ranged from 0.2460 to 0.4681 (mean 0.3523), and Shannon’s information index (I) ranged from 0.4004 to 0.6609 (mean 0.5202). Notably, the marker Sau031 exhibited the highest values for both H and I value. The PIC values for the 32 markers ranged from 0.5125 and 0.9109, with an average of 0.7371; the marker sau021 showed the highest PIC value. According to the classification criteria established by Wang et al. (2025), all PIC values fell within the range of 0.500 to 1.000, indicating a high level of polymorphism among the SSR markers developed in this study.

### 3.4 Cluster analysis based on genetic distance

**Figure 4:**
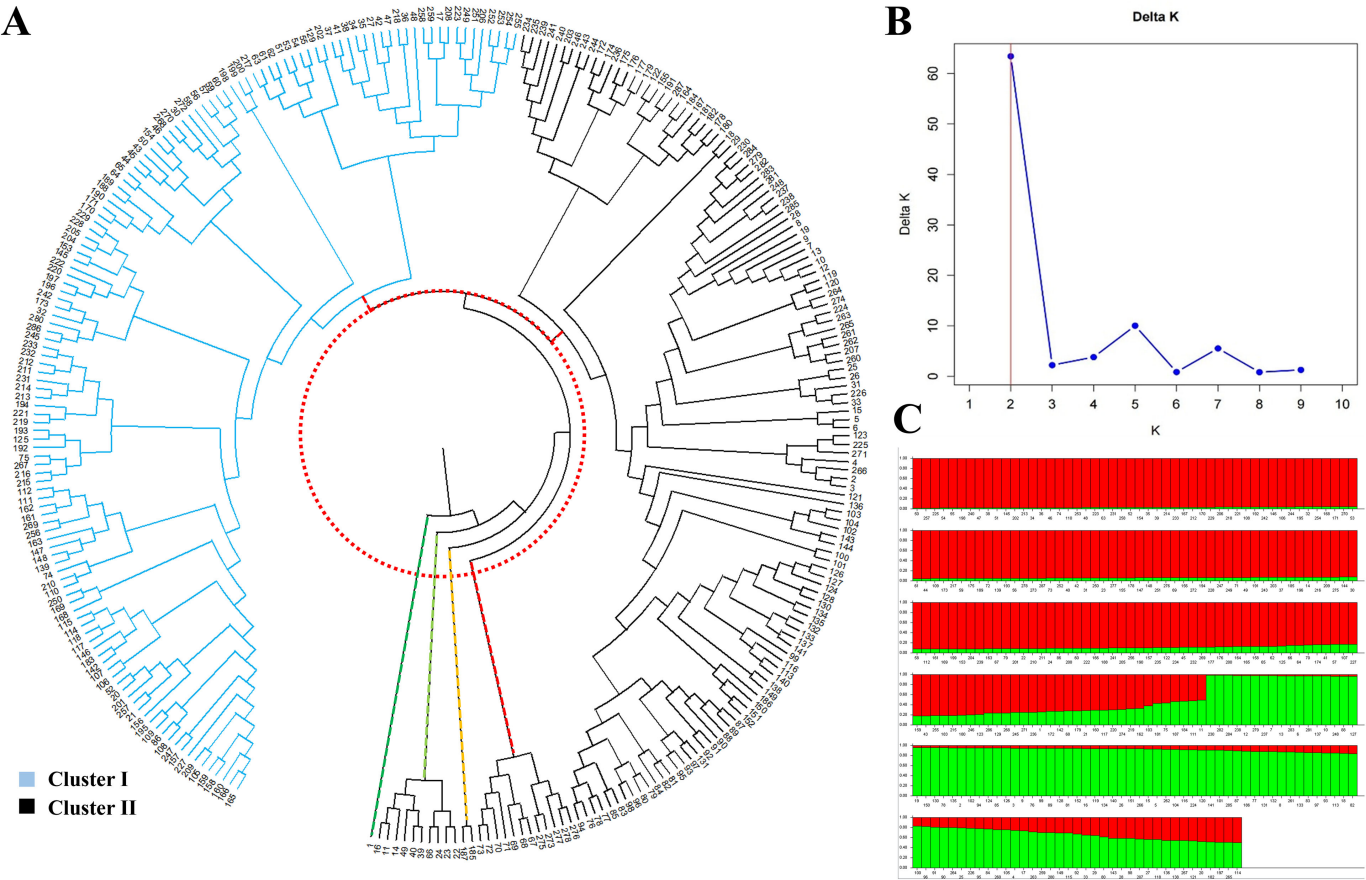
Genetic diversity analysis of 287 *Hemerocallis* germplasms. **(A)** UPGMA cluster analysis based on genetic distance; **(B)** Optimal number of population clusters (ΔK value); **(C)** Population genetic structure.

The UPGMA cluster analysis was performed using NTSYS software to calculate genetic distances of 287 *Hemerocallis* accessions, and a dendrogram was subsequently constructed with MEGA11 software. As shown in **Figure 4**, the 287 germplasm accessions were gathered as six primary groups at a genetic distancethreshold of approximately 0.01. The six groups could be further classified into two large cluster. The cluster I comprised 151 accessions, including *Hemerocallis* ‘Datong Huang Hua #1’, *Hemerocallis* ‘Chai Qiao Hua’, and *Hemerocallis* ‘Xi Ye Zi Hua’, representing the majority of the *Hemerocallis citrina* accessions in this study. The Cluster II consisted of 136 accessions, such as *Hemerocallis* ‘Dao Jian Hua #1’, *Hemerocallis* ‘Liu Lang Zhe’, and *Hemerocallis* ‘Zhi Jia Ge Luo Di Huang #1’, encompassing most *Hemerocallis fulva* accessions. The 32 SSR markers used in this study effectively discriminated all 287 *Hemerocallis* accessions.

### 3.5 Population genetic structure analysis

The STRUCTURE v2.3.4 software was used to analyze the population genetic structure of the 287 *Hemerocallis* accessions. Based on the ΔK method, the optimal number of clusters (K) was determined to be 2, corresponding to the maximum ΔK value (**Figure 4**). This result indicated that the 287 accessions can be divided into two distinct genetic groups (**Figure 4**). Group I comprised 186 accessions, including *Hemerocallis* ‘Autumn Red #1’, *Hemerocallis* ‘Little Wine Cup’, and *Hemerocallis* ‘Golden Doll #1’, representing the majority of the *Hemerocallis fulva* accessions. Group II consisted of the remaining 101 accessions, such as *Hemerocallis* ‘Datong Huang Hua #1’ and *Hemerocallis* ‘Guang Ling Huang Hua #1’, representing the majority of the *Hemerocallis citrina* accessions in this study.

### 3.6 Construction of an SSR-based data matrix for *Hemerocallis* germplasm accessions

From the 32 SSR markers, 13 markers with the highest PIC values were selected for data matrix construction, including sau021, sau013, sau009, sau014, sau034, sau043, sau046, sau024, sau007, sau032, sau041, sau042, sau006, with PIC values of 0.9109, 0.9100, 0.9054, 0.8808, 0.8714, 0.8668, 0.8455, 0.8251, 0.8235, 0.8178, 0.8164, 0.8049, and 0.7888, respectively. Amplification band patterns for each of these 13 markers were recorded for all 287 *Hemerocallis* accessions. The resulting genotype data were then combined with germplasm ID numbers and geographic origins to generate a unique molecular code for each accession (**Supplementary Table 4**).

### 3.7 Genetic linkage map construction for hybridization population of *Hemerocallis* ‘Liu Yue Hua’ × *Hemerocallis* ‘Frans Hals #1’

**Figure 5:**
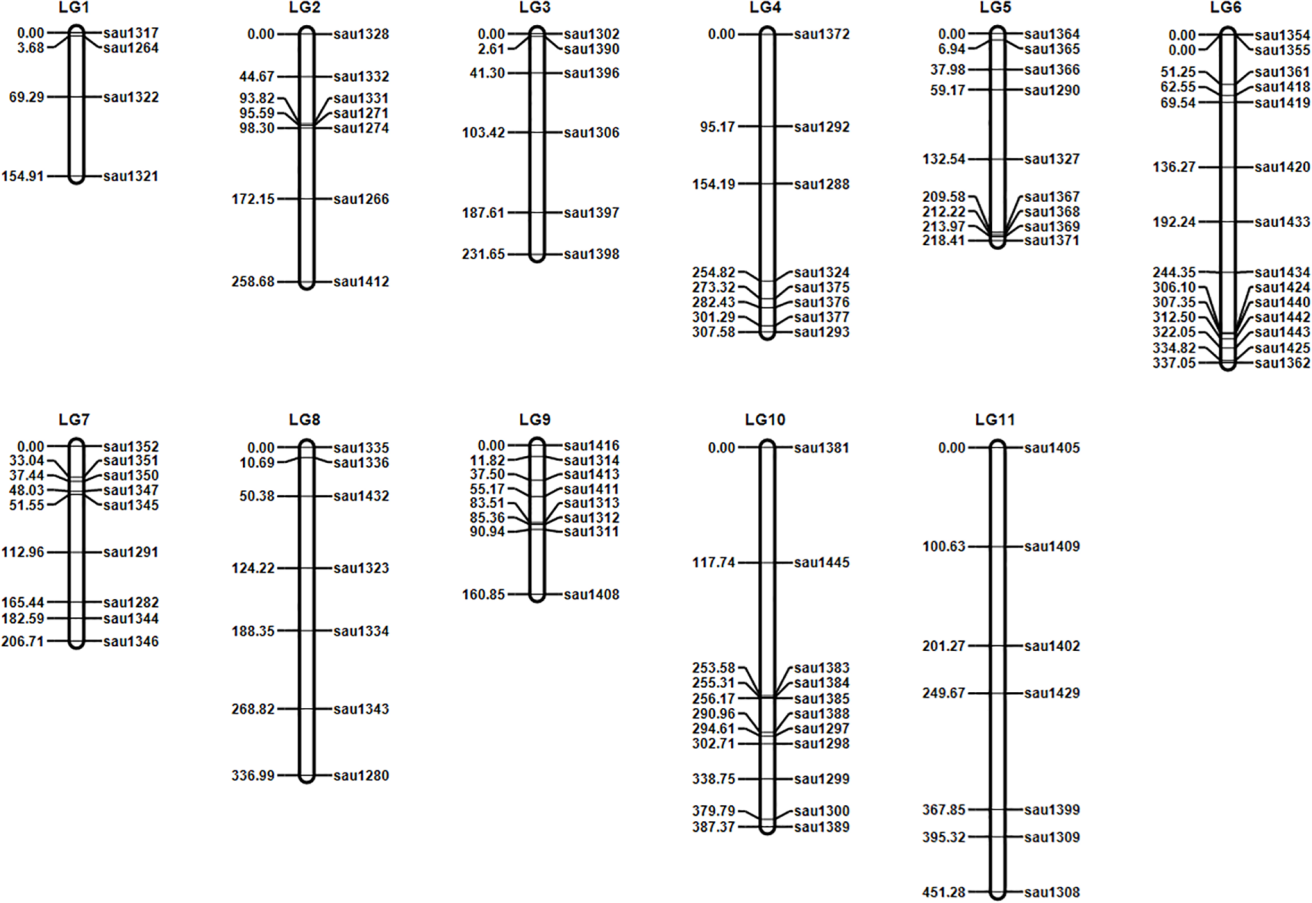
The genetic linkage map of *Hemerocallis* hybridization population of *Hemerocallis* ‘Liu Yue Hua’ × *Hemerocallis* ‘Frans Hals#1’.

The genetic linkage map of hybridization population for *Hemerocallis* ‘Liu Yue Hua’ × *Hemerocallis* ‘Frans Hals #1’ was constructed using genotyping data from 90 polymorphic SSR markers developed herein (**Supplementary Table 6**). This genetic map contained 90 SSR markers and 11 linkage groups (LG) (**Table 4**, **Figure 5**). The number of linkage groups corresponded to the diploid chromosome number of *Hemerocallis* (2n=2x=22). The linkage groups were numbered and ordered based on the physical positions of the *Hemerocallis* genome. The total map length was 3051.49 cM, with an average interval of 35.64 cM between adjacent markers. The LG6 contained the most markers of 14, whereas the LG1 had the fewest (4). The LG11 exhibited the greatest map length of 451.29 cM.

**Table 4:** Characteristics of genetic linkage map of *Hemerocallis*.

Linkage group	Num markers	Length (cM)	Mean distance	Maximum marker spacing (cM)
1	4	154.91	38.73	85.62
2	7	258.68	36.95	86.53
3	6	231.65	38.61	84.18
4	8	307.58	38.45	100.63
5	9	218.41	24.27	77.05
6	14	337.05	24.08	66.73
7	9	206.71	22.97	61.42
8	7	336.99	48.14	80.47
9	8	160.85	20.11	69.91
10	11	387.37	35.22	117.74
11	7	451.29	64.47	118.19
Total	90	3051.49	35.64	85.62

### 3.8 QTL mapping analysis

**Figure 6:**
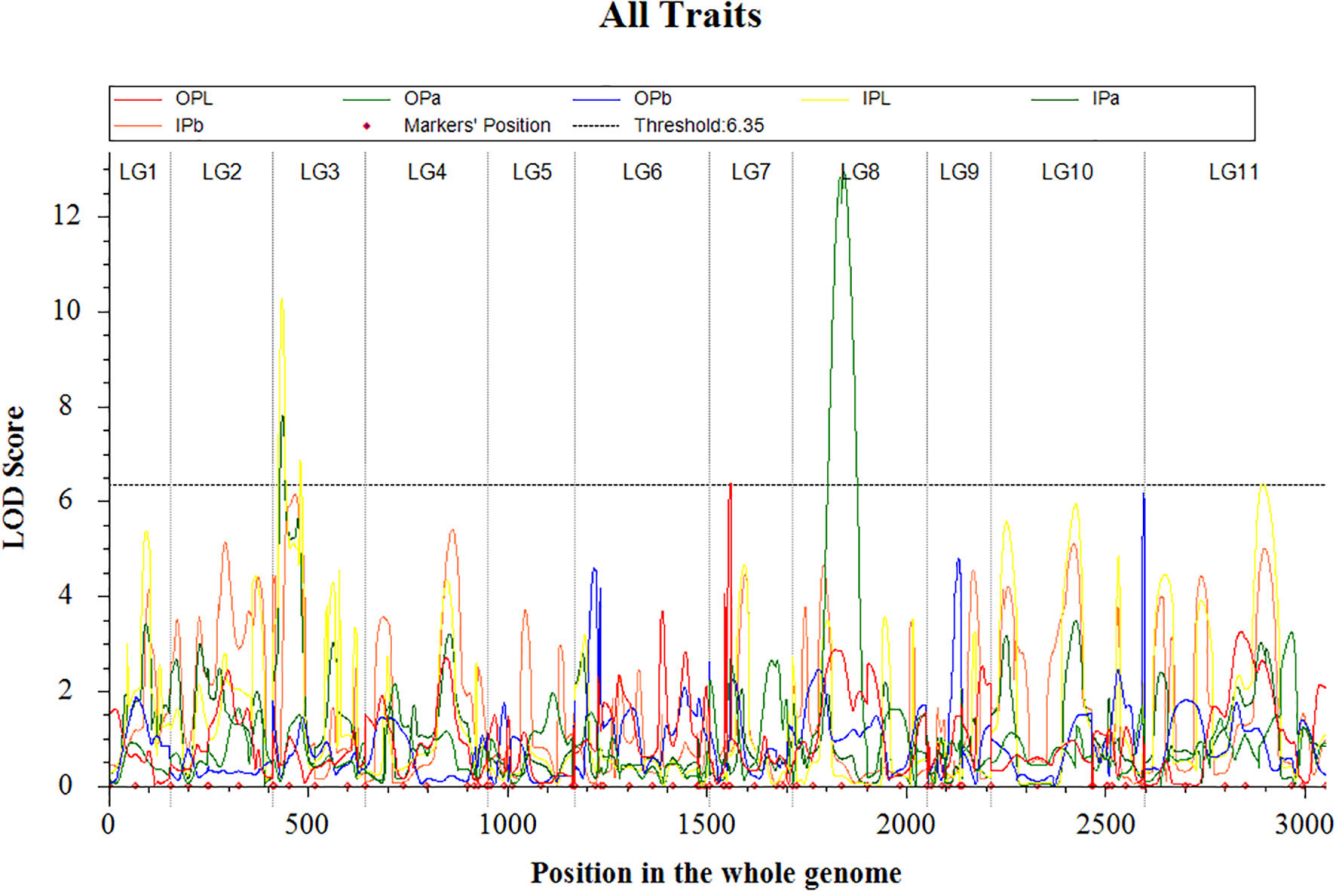
QTLs associated with petal color traits identified in the F_1_ hybridization population of *Hemerocallis* ‘Liu Yue Hua’ × *Hemerocallis* ‘Frans Hals#1’.

QTL mapping was performed for the inner and outer petal color traits in the F_1_ hybridization population derived from the cross between *Hemerocallis* ‘Liu Yue Hua’ and *Hemerocallis* ‘Frans Hals #1’, based on the genetic map constructed using 116 progenies. The phenotypic variation explained (PVE) by each QTL was estimated. A permutation test with 1000 iterations was conducted on the phenotypic data, and obtained a LOD threshold of 6.35 for petal color trait. A total of five QTLs were detected, distributed on LG3, LG7, LG8, and LG11. The LOD peak values ranged from 6.36 to 12.94, with the PVE values ranging from 8.45% to 12.95% (**Table 5**, **Figure 6**). For the outer petal color, two QTLs were identified. One QTL controlling the L* value of outer petal (*qOPL7.1*) was mapped on LG7, with a LOD peak of 6.36 and PVE of 23.49%. The QTL regulating the a* value of outer petal (*qOPa8.1*) was mapped on LG8, with a LOD peak of 12.94 and VE of 38.82%. For the inner petal color, four QTLs were detected. Three QTLs were associated with the L* value of inner petal. The *qIPL3.1* and *qIPL3.2* were both mapped on LG3, with LOD peaks of 10.26 and 6.85, and PVE of 9.85% and 10.08%, respectively. The *qIPL11.1* was located on LG11, with a LOD peak of 6.36 and PVE of 8.41%. Furthermore, the QTL controlling the a* value of inner petal (*qIPa3.1*) was mapped on LG3, with a LOD peak of 7.81, and PVE of 25.25%. Notably, *qIPL3.1*and *qIPa3.1* were located at the same position, and *qIPL3.2* was adjacent to the above two QTLs.

**Table 5:** The QTLs associated with petal color trait in hybridization population of *Hemerocallis* ‘Liu Yue Hua’ × *Hemerocallis* ‘Frans Hals #1’.

Trait	QTL	Linkage group	Position	Left marker	Right marker	LOD	PVE (%)	Physical distance (Mb)
L* value of outer petal	*qOPL7.1*	7	52	sau1345	sau1291	6.36	23.49	51.55~112.96
a* value of outer petal	*qOPa8.1*	8	130	sau1323	sau1334	12.94	38.82	124.22~188.35
L* value of inner petal	*qIPL3.1*	3	22	sau1390	sau1396	10.26	9.85	2.61~41.3
	*qIPL3.2*	3	68	sau1396	sau1306	6.85	10.08	41.3~103.42
	*qIPL11.1*	11	295	sau1429	sau1399	6.36	8.41	247.67~367.85
a* value of inner petal	*qIPa3.1*	3	24	sau1390	sau1396	7.81	25.26	2.61~41.3

## 4 Discussion

### 4.1 Characteristic of genome-wide SSR marker

Analysis of the genome-wide sequence data of *Hemerocallis* ‘Meng Zi Hua’ identified a total of 913,004 SSR loci, yielding an average SSR density of one locus per 4.14 kb. This frequency aligns with observations in numerous plant genomes, in which SSR loci typically occurred at intervals ranging from 1 to 10 kb (Parida et al., 2009). Six SSR repeat motifs were detected, involving from mononucleotide to hexanucleotide repeat motifs. The abundance of these repeat motifs varied considerably, with mononucleotide and dinucleotide repeats being predominant, constituting 30.42% and 42.25% of the total SSRs, respectively. In contrast, tri- to hexanucleotide repeats were less abundant, and the overall frequency of repeats decreased progressively with increasing motif length. This pattern was consistent with reports in the genomes of *Eucommia ulmoides* (Qing et al., 2022), *Arachis hypogaea* (Ma J, et al., 2020), and *Castanea mollissima* (Chu et al., 2025). Generally, smaller genomes tend to harbor a greater proportion of mononucleotide repeat motifs (Katti et al., 2001). The *Hemerocallis* ‘Meng Zi Hua’ genome, with a total length of approximately 3.6 Gb, contains a significant proportion of mononucleotide repeats relative to the *Eucommia ulmoides* genomes, which may reflect evolutionary variations accumulated over extended periods (Qing et al., 2022). Moreover, previous studies suggested that genomes rich in low-order repeat motifs may indicate an advanced stage of evolutionary development (Harr and Schlötterer, 2000). Collectively, these results suggest that the *Hemerocallis* genome has undergone considerable evolutionary progression and is characterized by a high mutation rate.

The A/T and AT/AT motif were predominant in *Hemerocallis* genome, accounting for 17.33% and 25.01% of total SSRs, respectively. Similar patterns have been observed in the genomes of apple (Larsen et al., 2018), sugarcane (Xu et al., 2025), and jute (Niyitanga et al., 2022). Conversely, in the genomes of *Erianthus arundinaceus* (Zeng et al., 2021), rice (Lawson and Zhang, 2006), and *Allium mongolicum* (Hu et al., 2017), the AG/CT motif is the most frequent. The repeat number of each SSR motif in the *Hemerocallis* genome also varied widely. SSRs with 12 to 20 repeats accounted for 36.73% of the total, and those SSRs with repeat number larger than 20 represented 18.83%. Mononucleotide and dinucleotide repeats were the main primary source of these high repeat number (>20) SSRs, accounting for 54,570 and 110,213 SSRs, respectively. Furthermore, the frequency of hexanucleotide repeats decreased as the repeat number increased. These findings suggested that the diversity in SSR distribution and frequency may be attributed to varying selective pressures encountered during evolutionary processes.

### 4.2 Genetic diversity and population structure of *Hemerocallis*

Genetic diversity is a critical goal in biodiversity conservation, as higher levels of genetic variation within a species enhance its adaptability to environmental changes. This genetic variability serves as a foundational element for plant conservation programs (Mantiquilla et al., 2022). In this study, the analysis of 287 *Hemerocallis* accessions using 32 SSR markers generated a total of 247 bands, with an average of 7.7 bands per marker. The PIC values for these 32 SSR markers were notably high, averaging 0.7371 and ranging from 0.5125 to 0.9109. Such elevated PIC values indicate polymorphism of the SSR markers and the diversity of the tested germplasm accessions, reflected the effectiveness of the SSR markers in this genetic diversity study. In a significantly contrast, a study by Misiukevicius et al. (2023), which also employed SSR markers to evaluate genetic diversity in daylily of *Hemerocallis* spp., reported a maximum PIC value of only 0.17 across 8 SSRs (Misiukevicius et al., 2023). This discrepancy suggested that the differences in the genetic background of the germplasm analyzed, which can significantly influence the observed PIC values. Even SSR markers with inherently high polymorphic potential may exhibit reduced PIC values in populations with low genetic diversity.

UPGMA cluster analysis groups accessions based on their genetic distance, thereby reflecting their genetic relatedness. Population structure analysis can reveal the ancestral origins, genetic composition, and the exchange of genetic information between different genotypes based on allele frequencies (Güney et al., 2018). In this study, UPGMA cluster analysis based on genetic distances calculated from the 32 SSR markers divided the 287 *Hemerocallis* accessions into six distinct groups. The six groups were further classified into two large cluster. Concurrently, population structure analysis of the same accessions was performed, and the optimal number of subpopulations (K value) was determined according to the ΔK value. The results revealed that ΔK was maximized at K = 2, suggesting that the 287 accessions could be broadly divided into two major genetic groups. The results of the two analysis methods were consistent. Furthermore, the clustering patterns observed in this study, based on 32 SSR markers, were similar with the findings of Li et al. (2021). These results indicate that although all accessions belong to the same genus, substantial genetic divergence exists among different germplasm of *Hemerocallis*. This information provides a valuable reference for the future classification and identification of *Hemerocallis* germplasm resources.

DNA fingerprinting is considered one of the simplest and most effective method for cultivar identification due to its multi-locus nature, high variability, and stable heritability (Chen et al., 2016). Markers with higher PIC values possess a greater capacity to discriminate between different varieties, making them suitable as core markers for DNA fingerprint. This approach enables the distinction of as many germplasms as possible using a minimal set of markers. For instance, Yan et al. (2024) developed 187 SSR markers and selected 16 core markers that could distinguish all tested mango germplasms, successfully constructed a fingerprint database for 229 germplasm accessions. Similarly, Liu et al. (2017) developed 36 SSR markers from genomic data and identified 5 core markers capable of completely distinguishing 80 tea germplasms. In this study, 13 markers with the highest PIC values were selected from a pool of 32 SSR markers. These markers were used to identify the germplasm accessions of *Hemerocallis* and recorded the amplification band, then a data matrix was constructed based on the resulting band profiles. The selected core markers are expected to reduce experimental costs and time in future large-scale identification of germplasm resources of *Hemerocallis*.

### 4.3 Construction of genetic linkage map and QTL mapping

The construction of genetic linkage maps and the mapping of QTLs mapping are fundamental approaches for important agronomic traits research and MAS breeding in crops. A prerequisite for genetic map construction is the establishment of an appropriate mapping population. In this study, an F_1_ hybridization population comprising 116 progenies was developed from a cross between *Hemerocallis* ‘Liu Yue Hua’ (female parent, with yellow inner and outer petals) and *Hemerocallis* ‘Frans Hals #1’ (male parent, with orange-red inner petals and yellow outer petals). The pronounced difference in petal color between the two parents made this hybridization population an ideal material for genetic mapping. The genetic map construction was performed using GACD software, a tool well-suited for genetic mapping in clonal F_1_ and double-cross populations. SSR markers were employed as the core markers for genetic map construction in this study. This choice was consistent with recent studies in other species, where SSR markers had served as core markers for linkage map construction in lavender (Georgieva et al., 2025), and nightlily (Hou et al., 2025), further highlighting the advantages of SSR markers, such as high polymorphism and ease of detection. For instance, Shi et al. (2025) constructed a genetic linkage map for freezing tolerance trait in almond using a combination of SRAP and SSR markers, integrating 110 polymorphic markers into 8 linkage groups. Similarly, for QTL mapping of panicle-related traits in foxtail millet, Li et al. (2025) adopted SSR and InDel markers, generating a map spanning 1545.5 cM with an average interval of 7.89 cM. In contrast to the single-marker (SSR) approach used in this study, those studies employed dual-marker systems. Such dual-marker approach can integrate the advantages of different marker types, covering genomic regions inaccessible to a single marker type and thereby improving map density and resolution.

In QTL mapping studies, loci with a relatively large PVE are generally referred to as major effect QTLs, while those with smaller PVE are called minor effect QTLs. A PVE threshold of 10% is commonly used to define major effect QTLs, this criterion widely applied in crops such as soybean (Tian et al., 2022), rose (Bourke et al., 2018), and sweet potato (Suematsu et al., 2025). In this study, a total of five QTLs were detected, distributed across the LG3, LG7, LG8, and LG11. The LOD peak scores for these QTLs ranged from 6.36 to 12.94, with corresponding PVE values ranging from 8.45% to 12.95%. It was important to note that the map constructed in this study based on a limited number of SSR markers, resulting in relatively large marker intervals. Furthermore, effective markers are difficult to design for certain genomic regions, leading to gaps in the map. These limitations may have reduced the accuracy of QTL mapping and hindered the discrimination of closely linked loci.

## 5 Conclusion

In this study, the genome-wide SSR markers based on the genome of *Hemerocallis* ‘Meng Zi Hua’ were developed, from which 913,004 SSR loci were identified. These loci were predominantly composed of mono- and dinucleotide repeats and featured AT-rich motifs. Two independent marker sets were subsequently designed for targeted applications. The first marker set comprised 32 polymorphic markers and was used to analyze the genetic diversity of 287 *Hemerocallis* germplasms accessions. Both UPGMA clustering and population structure analysis supported the division of these resources into two gene pools, *Hemerocallis citrina* accessions and *Hemerocallis fulva* accessions, and a fingerprint data matrix was constructed using 13 core markers from this set. These 32 markers provide a highly effective reference for future SSR molecular marker identification of *Hemerocallis* germplasm resources. The second marker set consisted of 90 parent-progeny polymorphic markers and was used to construct a genetic linkage map comprising 11 linkage groups. Five QTLs associated with petal color were detected with the constructed genetic map, among which *qIPL3.1* and *qIPa3.1* were located to the same position. These results indicate the potential utility of the constructed genetic linkage map for mapping botanical traits in *Hemerocallis* germplasm. Future efforts should aim to increase the marker density of this map to improve its resolution for gene localization. In particular, fine mapping of the two key QTLs identified should be conducted to screen candidate genes highly linked to petal color in *Hemerocallis*. Collectively, these genome-wide SSR markers significantly expand the molecular resources available for *Hemerocallis*. The targeted application of these two marker sets facilitates germplasm identification, MAS breeding, and genetic improvement of *Hemerocallis*.

## Data Availability

The original contributions presented in the study are included in the article/**supplementary material.** Further inquiries can be directed to the corresponding authors.
