# Supplementary figures and images for: Genome-wide SSR marker development and application in molecular breeding of Hemerocallis
Source: Front Plant Sci. 2026 Mar 25;17:1740316. doi: 10.3389/fpls.2026.1740316 (PMC13057510; doi:10.3389/fpls.2026.1740316)

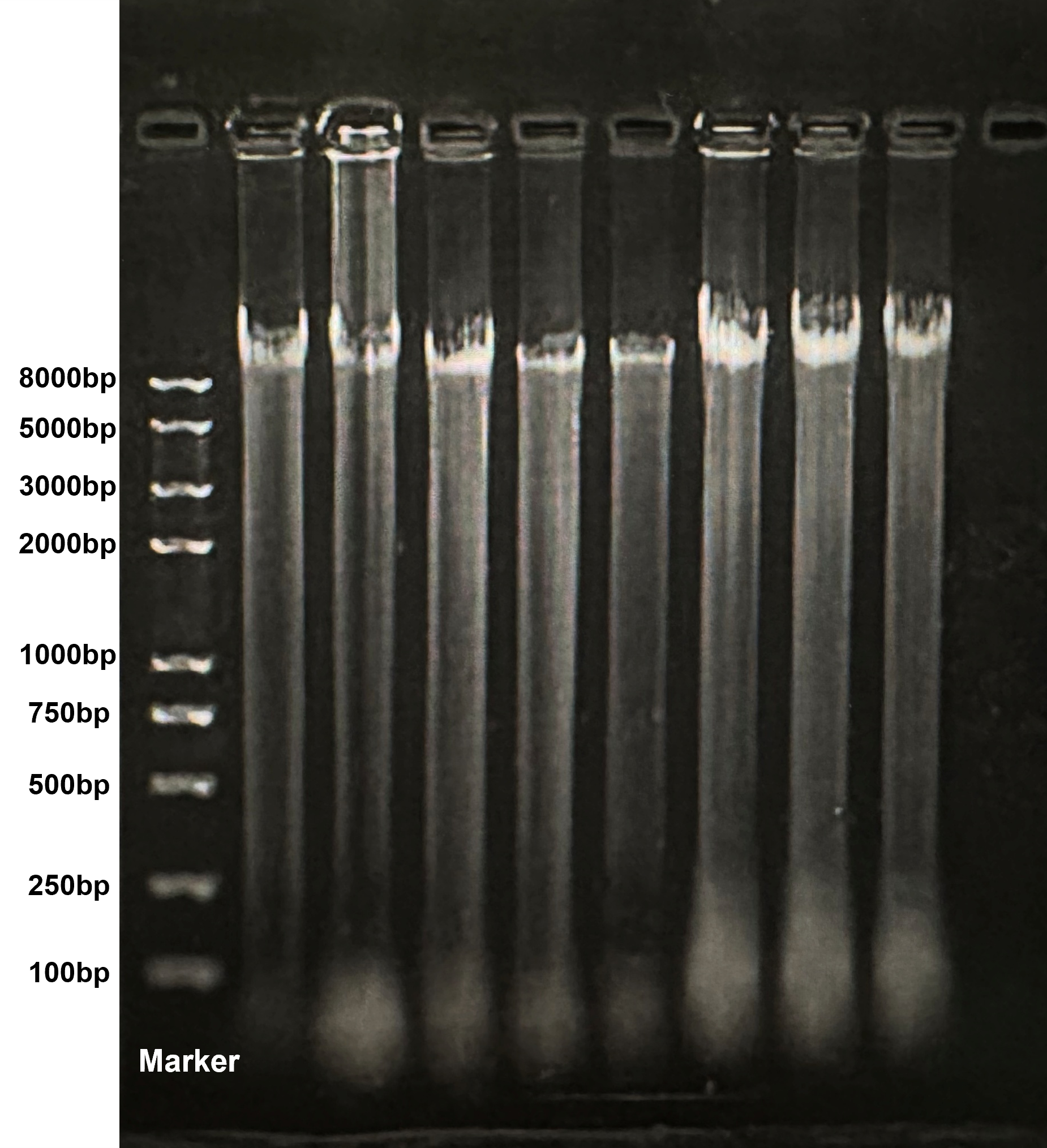

Supplement: Supplementary file 1 [file Image1.tif]
